# Application of Phosphonium Ionic Liquids as Ion Carriers in Polymer Inclusion Membranes (PIMs) for Separation of Cadmium(II) and Copper(II) from Aqueous Solutions

**DOI:** 10.1007/s10953-015-0413-2

**Published:** 2015-11-12

**Authors:** Beata Pospiech

**Affiliations:** Department of Chemistry, Czestochowa University of Technology, Armii Krajowej 19, 42-200 Czestochowa, Poland

**Keywords:** Cadmium(II), Copper(II), Cyphos IL 101, Cyphos IL 104, Polymer inclusion membrane (PIM)

## Abstract

Facilitated transport through polymer inclusion membranes (PIMs) is a promising method for simultaneous separation and removal of valuable and toxic metal ions from aqueous solutions. Recently, ionic liquids (ILs) have been used as extracting agents for metal ions due to their unique physicochemical properties. This paper presents research on the facilitated transport of cadmium(II) and copper(II) ions from aqueous chloride solutions through PIMs with phosphonium ILs as new selective ion carriers. Cellulose triacetate membranes containing *o*-nitrophenyl octyl ether (ONPOE) as a plasticizer and Cyphos IL 101 [trihexyl(tetradecyl)phosphonium chloride] or Cyphos IL 104 [trihexyl(tetradecyl)phosphonium bis(2,4,4-trimethylpentyl)phosphinate] as the ion carriers have been prepared and applied for investigations. Cd(II) ions were transported preferably from hydrochloric acid solutions containing Cu(II) ions through the PIMs. Higher selectivity coefficient of Cd(II) over Cu(II) (*S*_Cd/Cu_) from 0.1 mol·dm^−3^ hydrochloric acid was obtained for PIM with Cyphos IL 104 as the ion carrier. The influence of HCl and NaCl concentrations in the source phase on metal ion transport across PIM doped with Cyphos 104 was studied. It was found that the initial fluxes of Cd(II) and Cu(II) increase with increasing chloride ions concentration in the source phase. The selectivity coefficient for Cd(II) over Cu(II) decreases with increasing HCl concentration in the source phase. The results suggest that the separation system presented in this paper can be useful for the removal of Cd(II) from acidic chloride solutions in the presence of Cu(II).

## Introduction

Techniques for the separation of metal ions from industrial and waste solutions are extremely important in industrial applications. Increasing demand on metal production leads to research into efficient and economical methods for separation and recovery of metal ions [1, 2]. Cadmium occurs as a minor constituent of base metal ores. Leach liquors of ores contain many valuable metals. Several technologies are used for selective removal of metals, including precipitation, solvent extraction, ion exchange, etc. [3]. Transport of metal ions from aqueous dilute solutions through liquid membranes was established as an alternative to other separation methods. Polymer inclusion membranes (PIMs) are a type of liquid membrane (LMs). PIMs usually contains a polymer support, an ion carrier and a plasticizer. The base polymer provides mechanical strength to the membrane. Plasticizer improves the flexibility and transport properties of the membrane. Moreover, plasticizer is also a solvent for an ion carrier, which is an extracting agent. It is worth highlighting that the volumes of organic solutions (carriers, plasticizers, ion carriers) which are needed for synthesis of PIMs are very low [4, 5]. *O*-nitrophenyl octylether (ONPOE) was used as plasticizer in this study because this compound shows remarkable physicochemical properties, including: a high dielectric constant, low solubility in water and low toxicity [6]. Moreover it acts as a good organic solvent for carrier molecules and improves the membranes softness and flexibility [7]. The choice of plasticizer influences the separation properties of the prepared membranes because the transport flux of metal ions depends strongly on the chemical nature of the plasticizer [8]. The selectivity and efficiency of transport processes depend on the polarity and viscosity of the plasticizers. Increasing the viscosity of the plasticizer decreases the rate of transport. Plasticizers with high polarity can be used preferentially as solvents in membranes [9]. Kebiche-Senhadji et al. [10] reported that the dielectric constant influences the balance between the efficiency of association and disassociation for the uptake of the metal ions by the ion carrier and its release from the complex metal-ion carrier at the receiving interface.

Cellulose triacetate (CTA) based membranes show good potential for industrial separation techniques due to high chemical stability and mechanical resistance, ease of synthesis, reduced consumption of reagents and high process selectivity [11, 12].

The transport of heavy metals from aqueous solutions, across PIMs, has been the subject of many investigations. PIMs, which are used for removal of metal ions contain different compounds as ion carriers, such as: di(2-ethylhexyl) phosphoric acid (D2EHPA) [11–13], thiophosphinic oxide (TOPO) [11, 14], Cyanex 301 [di(2,4,4-trimethylpentyl) dithiophosphinic acid] [15], Cyanex 471X (triisobutylphosphine sulphide) [16, 17], Aliquat 336 (trioctylmethylammonium chloride) [18–21], TOA (trioctylamine) [3], TIOA (triisoctylamine) [22], etc.

Recently, ionic liquids (ILs) have been used as extractants or ion carriers for metal ions from aqueous solutions. ILs exhibit many unique properties, such as: negligible vapor pressure, high thermal stability, non-flammability and good extractability for various organic compounds as well as for metal ions [23, 24]. Room temperature ionic liquids (RTILs) are an alternative to replace organic solvents because they are able to dissolve both polar and non-polar compounds. The important advantage of ILs is their negligible vapor pressure, which results in lower emissions as well as their good chemical and thermal stabilities. Therefore, ionic liquids are considered as environmental friendly as well as low toxicity green solvents solvents as compared to volatile organic solvents [25–27].

Nowadays, an increasing interest in technologies of PIMs containing ILs can be observed [28–31]. Cyphos IL 101 and Cyphos IL 104 were used as extractants and ion carriers of Zn(II) and Fe(II) from aqueous chloride solutions [28, 29]. Zn(II) extraction from the feed containing 5 g·dm^−3^ exceeds 85 %; meanwhile, Fe(II) extraction was only 20 % [28]. Transport process of Zn(II) and Fe(III) ions through a PIM with Cyphos IL 101 was relatively slow. Conversely, much more efficient transport through a PIM was observed for Fe(III) than Zn(II) [29]. Kogelnig et al. [30] reported results of the transport of Zn(II) from aqueous solution using PIMs based on poly(vinyl chloride) containing Cyphos IL 101. Zn(II) was efficiently extracted from 5 mol·dm^−3^ HCl through membrane containing 30 wt.% Cyphos 101, whereas back-extraction was successfully achieved with 1 mol·dm^−3^ H_2_SO_4_ [29]. Removal of Cr(VI) ions from hydrochloric acid solutions using PIMs with Cyphos 104 has been studied by Guo et al. [31]. Polyvinylidene fluoride (PVDF) was used as polymeric support of these membranes and [C_8_mim][BF_4_] as plasticizer. The maximum permeability coefficient (*P*) was 9.64 μm·s^−1^.

Separation of Cd(II) and Cu(II) from hydrochloric acid solutions by transport through PIMs with phosphonium ionic liquids as ion carriers has not been reported. The aim of the present work is to study the facilitated transport of Cd(II) and Cu(II) across PIMs with Cyphos IL 101 and Cyphos IL 104. The permeation of the metal ions is investigated as a function of various experimental variables: concentration of hydrochloric acid and sodium chloride in the source phase, carrier concentration in the membrane, etc. The transport selectivity of Cd(II) and Cu(II) from hydrochloric acid and sodium chloride solutions was also investigation.

## Experimental

### Reagents

Cadmium(II) chloride (CdCl_2_·2.5H_2_O, 99 %), copper(II) chloride (CuCl_2_·2H_2_O, 99 %), sodium chloride (NaCl), hydrochloric acid (HCl), and sulfuric acid (H_2_SO_4_) were of analytical grade and were purchased from POCh (Gliwice, Poland). Aqueous solutions were prepared with deionized water.

Trihexyl(tetradecyl)phosphonium chloride (Cyphos^®^ IL 101) and trihexyl(tetradecyl)phosphonium bis(2,4,4-trimethylpentyl)phosphinate (Cyphos^®^ IL 104) of 95 % purity were purchased from Cytec Canada Industries Inc.; CTA, dichloromethane, toluene, and *o*-nitrophenyl octyl ether (ONPOE) were of analytical reagent grade (Aldrich) and used without further purification. The density of the plasticizer, ONPOE, is 1.041 g·cm^−3^.

### Polymer Inclusion Membrane

The membranes were prepared as described elsewhere [16–18]. Solutions of CTA, the ion carrier (Cyphos IL 101, Cyphos IL 104), and the plasticizer [*o*-nitrophenyl octyl ether (ONPOE)] were prepared in dichloromethane. A portion of this solution was poured on a flat-bottom glass Petri dish which was kept on a leveled surface. The organic solvent was allowed to evaporate for 12 h. The resultant membrane was peeled off from the Petri dish and this membrane was washed with water and equilibrated in distillated water before use.

### Transport Experiments

Transport experiments were carried out in the experimental setup of PIM system described in the earlier papers [16–18]. The volumes of source and receiving phases were 100 cm^3^, respectively. The effective membrane area, which was exposed to both phases, was 12.56 cm^2^. Both the source and receiving aqueous phases were stirred using a magnetic stirrer. To transport Cd(II) and Cu(II) across the PIM, a membrane module was used to which both aqueous phases were pumped with a peristaltic pump (PP1B-05A type, Zalimp, Poland) working at a speed of 100 cm^3^·min^−1^ from tanks containing source and receiving phases, respectively. The schematic diagram of the experimental setup of PIM system has been shown in the earlier paper [16]. The permeation of metal ions was monitored by sampling of the source phase, which was analyzed by an atomic absorption spectrophotometer (Solaar 939, Unicam).

The kinetics of PIM transport processes is described by a first-order reaction in metal ion concentration [16, 17]:

1 $$ \ln \left( {\frac{c}{{c_{i} }}} \right) = - kt $$

where *c* is the metal ion concentration in the source phase at some given time, *c*_*i*_ is the initial metal ion concentration in the source phase, *k* is the rate constant, and *t* is the time of transport.

To calculate the *k* value, a plot of ln(*c/c*_*i*_) versus time was prepared. The rate constant value for the duplicate transport experiment was then averaged and standard deviation was calculated. The permeability coefficient *(P)* was calculated as follows:

2 $$ P = \frac{V}{A}k $$

where *V* is volume of the aqueous source phase, and *A* is the effective area of membrane.

The initial flux *(J*_*i*_*)* was determined as:

3 $$ J_{i} = P \cdot c_{i} $$

The selectivity coefficient (*S*) is defined as the ratio of initial fluxes for M_1_ and M_2_ metal ions, respectively:

4 $$ S = \frac{{J_{{i,M_{1} }} }}{{J_{{i,M_{2} }} }} $$

The recovery factor (*RF*) of metal ions from the source phase into the receiving phase was calculated as:

5 $$ RF = \frac{{c_{i} - c}}{{c_{i} }} \cdot 100\% $$

### Solvent Extraction

Solvent extraction was carried out as reported earlier [18, 32]. Equal volumes of organic and aqueous phases (phase volume ratio O/A = 1) were mechanically shaken for 20 min at 25 ± 2 °C. After settling, the aqueous phase was separated from the organic phase. The equilibrium pHs of the aqueous phases were measured with an Elmetron CX-731 pH-meter equipped with a glass electrode (Hydromet, Poland). Metal ion concentrations in the aqueous phases were determined with an AAS Solaar 939 (Unicam) atomic absorption spectrophotometer. The concentrations of metal ions in the aqueous phases were determined before and after solvent extraction to allow calculation of the extraction efficiency and after stripping to calculate the stripping efficiency. The concentrations of metals in the organic phases were calculated by mass balance. Each experiment was carried out three times and the error did not exceed 5 %. Distribution ratio (*D*) and extraction efficiency (%*E*) are defined as follows:

6 $$ D = \frac{{\left[ {\text{M}} \right]_{\text{org}} }}{{\left[ {\text{M}} \right]_{\text{aq}} }} $$

7 $$ \% E = \frac{D}{D + 10} \cdot 100\;\% $$

where [M]_org_ and [M]_aq_ denote metal concentrations in the organic and aqueous phases after solvent extraction, respectively.

The electivity coefficient (*S*_M1/M2_) for M_1_ over M_2_ was calculated as follows:

8 $$ S_{M1/M2} = \frac{{D_{M1} }}{{D_{M2} }} $$

### Stripping Experiments

To evaluate the stripping efficiency of sulfuric acid, the extraction solvent was first contacted with an aqueous solution containing 0.01 mol·dm^−3^ Cd(II) and 0.01 mol·dm^−3^ Cu(II) in 0.1 mol·dm^−3^ HCl with 0.1 mol·dm^−3^ Cyphos IL 101 and 0.1 mol·dm^−3^ Cyphos IL 104, respectively, at a phase volume ratio O:A = 1. After settling, the organic phase was separated from aqueous phase. The organic phase was then put in contact with the stripping solution (sulfuric acid solution) at phase volume ratio O:A = 1 at 25 ± 2 °C. After shaking, the organic and aqueous phases were separated and the metal concentrations in the aqueous phases were determined by AAS to calculate the stripping efficiency.

## Results and Discussion

### Solvent Extraction of Cd(II) and Cu(II) from HCl Solutions

**Fig. 1:**
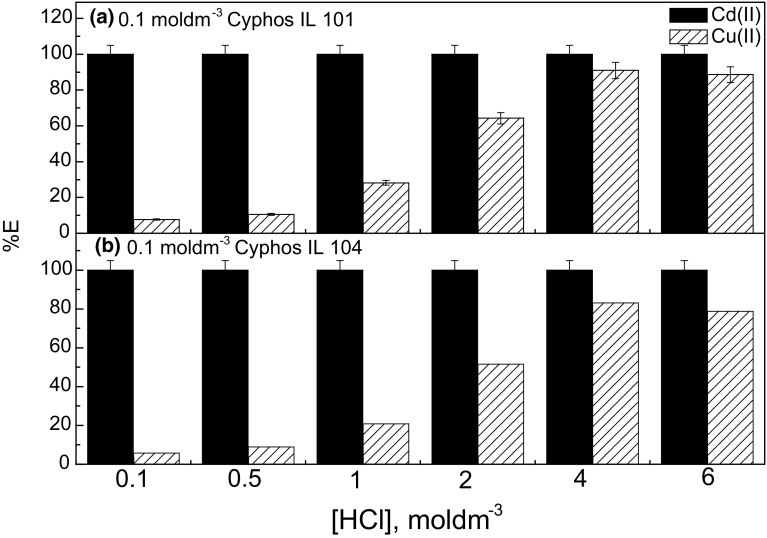
Extraction efficiency of Cd(II) and Cu(II) as a function of HCl concentration by ILs. **a** 0.1 mol·dm^−3^ Cyphos IL 101 in toluene; **b** 0.1 mol·dm^−3^ Cyphos IL 104 in toluene. Aqueous phase: 0.01 mol·dm^−3^ Cd(II) and 0.01 mol·dm^−3^ Cu(II) in HCl solutions

The selective extraction of Cd(II) and Cu(II) from hydrochloric acid solutions is difficult because both metal ions exist as anionic chloride complexes at high chloride concentration. Copper chloride complexes seem to have the same extractability as cadmium chloride complexes. The phosphonium ILs, such as: Cyphos IL 101 and Cyphos IL 104 have been chosen for the separation process of Cd(II) and Cu(II) because these salts differ by anions that can affect extraction properties of these ILs. Cyphos IL 101 contains a hydrophilic chloride anion, while Cyphos IL 104 is a derivative of Cyanex 272 and contains bis(2,4,4-trimethylpentyl)phosphinate anion. To better understand the transport behavior of Cd(II) and Cu(II) from hydrochloric acid solutions through PIMs with Cyphos IL 101 and Cyphos Il 104 as the ion carriers, it was necessary to perform solvent extraction studies with the ionic liquids as extractants. To investigate the influence of hydrochloric acid concentration in the aqueous phase on the extraction efficiency of metal ions, the experiments were performed at different concentration of this acid. The concentration of HCl varied from 0.1 to 6 mol·dm^−3^. Figure 1 shows the effect of HCl concentration on the extraction efficiency of Cd(II) and Cu(II) by 0.1 mol·dm^−3^ Cyphos IL 101 and 0.1 mol·dm^−3^ Cyphos IL 104 in toluene, respectively. As can be seen from this figure, the extraction efficiency of Cd(II) was very high at all HCl concentrations with Cyphos IL 101 and Cyphos IL 104 and reached 99.9 % independent of the HCl concentration. The extraction efficiency of Cu(II) increased with HCl concentration increasing up to 5 mol·dm^−3^ HCl, and then decreased for higher HCl concentrations (Fig. 1). As can be observed, the highest extraction efficiency of Cu(II) was 90.1 vol% from 5 mol·dm^−3^ HCl by 0.1 mol·dm^−3^ Cyphos IL 101 in toluene.

**Fig. 2:**
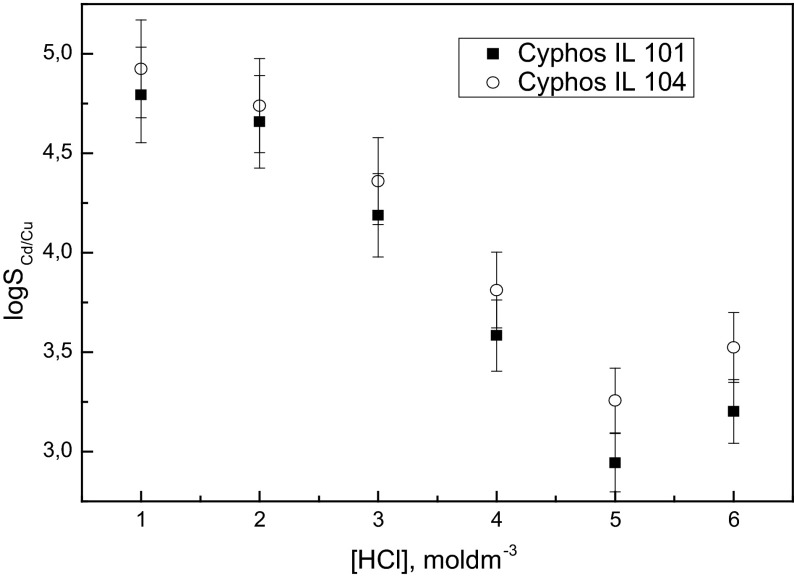
Logarithm of selectivity coefficient (log_10_ *S*_Cd/Cu_) of Cd(II) over Cu(II) versus HCl concentration. Aqueous phase: 0.01 mol·dm^−3^ Cd(II), 0.01 mol·dm^−3^ Cu(II) in HCl; organic phase: 0.1 mol·dm^−3^ Cyphos IL 101 and 0.1 mol·dm^−3^ Cyphos IL 104 in toluene

The logarithm of the selectivity coefficient (log_10_*S*_Cd/Cu_) obtained with Cyphos 101 as well as by 0.1 mol·dm^−3^ Cyphos 104 in toluene decreases when the HCl concentration increases in the range from 0.1 to 5 mol·dm^−3^, as is shown in Fig. 2. The logarithm of the selectivity coefficient (log_10_*S*_Cd/Cu_) slightly increased for both extractants at 6 mol·dm^−3^ HCl. The selectivity coefficients for Cd(II) over Cu(II) with Cyphos 104, depending on HCl concentration, are higher than those obtained with Cyphos 101. We can find in the literature that quaternary phosphonium salts can extract both neutral and anionic complexes [28, 29]. Extraction of Cd(II) and Cu(II) by Cyphos IL 101 and Cyphos IL 104 probably proceeds by different mechanisms depending on the acid and chloride concentrations in the aqueous phase. The extraction efficiency of Cd(II) by Cyphos IL 104 is very high and independent of HCl concentration in the aqueous solutions. We know that in chloride solutions cadmium(II) forms the cationic chlorocomplex CdCl^+^ as well as stable complexes of the type CdCl_2_ and ${\text{CdCl}}_{3}^{ - }$ at high chloride ions concentrations (Table 3). The extraction results suggest that not only an anion exchange mechanism of extraction occurs by Cyphos IL 104, because Cd(II) does not form stable anionic chlorocomplexes in 0.1 mol·dm^−3^ HCl. The proposed mechanism is given by the following equations:

Cyphos IL 104

9 $$ {\text{Cd}}_{\text{aq}}^{2 + } + 2{\text{Cl}}_{\text{aq}}^{ - } + 2{\text{R}}_{3} {\text{R}}'{\text{PA}}\underset{{}}{\overset{{}}{\rightleftharpoons}}2{\text{R}}_{3} {\text{R}}'{\text{PCl}}_{\text{org}} + {\text{CdA}}_{{2{\text{ org}}}} $$

10 $$ {\text{CdCl}}_{\text{aq}}^{ + } + {\text{Cl}}_{\text{aq}}^{ - } + 2{\text{R}}_{3} {\text{R}}'{\text{PA}}_{\text{org}} \underset{{}}{\overset{{}}{\rightleftharpoons}}2{\text{R}}_{3} {\text{R}}'{\text{PCl}}_{\text{org}} + {\text{CdA}}_{{2{\text{ org}}}} $$

11 $$ {\text{CdCl}}_{{2{\text{ org}}}} + 2{\text{R}}_{3} {\text{R}}'{\text{PA}}_{\text{org}} \underset{{}}{\overset{{}}{\rightleftharpoons}}2{\text{R}}_{3} {\text{R}}'{\text{PCl}}_{\text{org}} + {\text{CdA}}_{{2{\text{ org}}}} $$

12 $$ {\text{H}}_{\text{aq}}^{ + } + {\text{CdCl}}_{{3{\text{ aq}}}}^{ - } + {\text{R}}_{3} {\text{R}}'{\text{PA}}_{\text{org}} \underset{{}}{\overset{{}}{\rightleftharpoons}}{\text{R}}_{3} {\text{R}}'{\text{PCdCl}}_{{3{\text{ org}}}} + {\text{HA}}_{\text{org}} $$

13 $$ {\text{CuCl}}_{{2{\text{ aq}}}} + 2{\text{R}}_{3} {\text{R}}'{\text{PA}}_{\text{org}} \underset{{}}{\overset{{}}{\rightleftharpoons}}2{\text{R}}_{3} {\text{R}}'{\text{PCl}}_{\text{org}} + {\text{CuA}}_{{2{\text{ org}}}} $$

14 $$ 2 {\text{H}}_{\text{aq}}^{ + } + {\text{CdCl}}_{{4{\text{ aq}}}}^{2 - } + {\text{R}}_{3} {\text{R}}'{\text{PA}}_{\text{org}} \underset{{}}{\overset{{}}{\rightleftharpoons}}{\text{R}}_{3} {\text{R}}'{\text{PHCdCl}}_{{4{\text{ org}}}} + {\text{HA}}_{\text{org}} $$

where A denotes the bis(2,4,4-trimethylpentyl)phosphinate anion, subscripts (aq) and (org) stand for the aqueous and organic phases, respectively.

Cyphos IL 101

15 $$ {\text{CdCl}}_{\text{aq}}^{ + } + {\text{Cl}}_{\text{aq}}^{ - } + 2{\text{R}}_{3} {\text{R}}'{\text{PCl}}_{\text{org}} \underset{{}}{\overset{{}}{\rightleftharpoons}}\left( {{\text{R}}_{3} {\text{R}}'{\text{P}}} \right)_{2} {\text{CdCl}}_{{ 4 {\text{ org}}}} $$

16 $$ {\text{CdCl}}_{{3{\text{ aq}}}}^{ - } + {\text{R}}_{3} {\text{R}}'{\text{PCl}}_{\text{org}} \underset{{}}{\overset{{}}{\rightleftharpoons}}{\text{R}}_{3} {\text{R}}'{\text{PCdCl}}_{{3{\text{ org}}}}^{ - } + {\text{Cl}}_{\text{aq}} $$

17 $$ {\text{CdCl}}_{{4{\text{ aq}}}}^{2 - } + 2{\text{R}}_{3} {\text{R}}'{\text{PCl}}_{\text{org}} \underset{{}}{\overset{{}}{\rightleftharpoons}}\left( {{\text{R}}_{3} {\text{R}}'{\text{P}}} \right)_{2} {\text{CdCl}}_{{4{\text{ org}}}}^{2 - } + 2{\text{Cl}}_{\text{org}}^{ - } $$

18 $$ {\text{CdCl}}_{{2{\text{ aq}}}} + 2{\text{R}}_{3} {\text{R}}'{\text{PCl}}_{\text{org}} \underset{{}}{\overset{{}}{\rightleftharpoons}}\left( {{\text{R}}_{3} {\text{R}}'{\text{P}}} \right)_{2} {\text{CdCl}}_{{4{\text{ org}}}} $$

19 $$ {\text{CuCl}}_{{2{\text{ aq}}}} + 2{\text{R}}_{3} {\text{R}}'{\text{PCl}}_{\text{org}} \underset{{}}{\overset{{}}{\rightleftharpoons}}\left( {{\text{R}}_{3} {\text{R}}'{\text{P}}} \right)_{2} {\text{CuCl}}_{{4{\text{ org}}}}^{2 - } $$

20 $$ {\text{CuCl}}_{{4{\text{ aq}}}}^{2 - } + 2{\text{R}}_{3} {\text{R}}'{\text{PCl}}_{\text{org}} \underset{{}}{\overset{{}}{\rightleftharpoons}}\left( {{\text{R}}_{3} {\text{R}}'{\text{P}}} \right)_{2} {\text{CuCl}}_{{4{\text{ org}}}}^{2 - } + 2{\text{Cl}}_{\text{org}}^{ - } $$

The difference in the extraction behavior of Cd(II) and Cu(II) can be attributed to the fact that in chloride solutions cadmium(II) forms mainly stable complexes of the type CdCl_2_ and ${\text{CdCl}}_{3}^{ - }$ at high chloride ions concentrations, while copper(II) exists mainly as CuCl_2_ and ${\text{CuCl}}_{4}^{2 - }$ [33, 34]. Further investigations are necessary to confirm these suggested reactions of Cd(II) and Cu(II) with the phosphonium ionic liquids.

### Stripping

Sulfuric acid solutions were tested to strip Cd(II) and Cu(II) from the loaded organic phases at phase volume ratio A:O = 1. Recovery results for various sulfuric acid concentrations as the stripping solutions are presented in Table 1. Examination of this table indicates that sulfuric acid leads to high Cd(II) and Cu(II) stripping efficiency but the stripping of Cd(II) from the organic phase is not effective with dilute acid. It can be observed that increasing the H_2_SO_4_ concentration causes a significant increase in the Cd(II) and Cu(II) stripping efficiency. The most efficient stripping phase of the studied metal ions from Cyphos IL 101 and Cyphos IL 104 seems to be 1 mol·dm^−3^ H_2_SO_4_. The stripping of Cd(II) and Cu(II) was found to be above 82 and 92 %, respectively, in a single contact by 1 mol·dm^−3^ H_2_SO_4_ for both extractants. Thus, 1 mol·dm^−3^ H_2_SO_4_ was chosen as the receiving solution in the transport process through PIMs. The bonds in the metal–ionic liquid complexes in the organic phase are very strong but the application of 1 mol·dm^−3^ H_2_SO_4_ allows the effective removal of Cd(II) from the organic phase into the aqueous phase. This stripping solution strongly attracts Cd(II) from the ILs; therefore it seems to be a suitable stripping agent in this process.

**Table 1:** Cd(II) and Cu(II) stripping efficiencies by sulfuric acid from 0.1 mol·dm^−3^ Cyphos IL 101 and 0.1 mol·dm^−3^ Cyphos IL 104 in toluene previously loaded by contacting the extraction solvent with an aqueous phase containing 0.01 mol·dm^−3^ Cd(II) and 0.01 mol·dm^−3^ Cu(II) in 0.1 mol·dm^−3^ HCl

Stripping solution (H_2_SO_4_) (mol·dm^−3^)	Stripping efficiency of Cd(II) (%)	Stripping efficiency of Cu(II) (%)	Stripping efficiency of Cd(II) (%)	Stripping efficiency of Cu(II) (%)
	Cyphos IL 101		Cyphos IL 104	
0.1	28.4	48.6	29.6	53.0
0.2	35.2	52.6	38.8	57.4
0.5	46.8	83.2	51.5	85.7
1.0	82.4	92.9	83.1	93.5

Phase volume ratio O/A = 1; *t* = 25 °C

### Transport of Cd(II) and Cu(II) Through PIMs with Cyphos IL 101 and Cyphos IL 104

Recently, we have observed that ionic liquids play an increasingly important role in separation techniques, including extractions and membrane processes and are frequently used for separation of metal ions. The membranes used in the present work for studying the transport of Cd(II) and Cu(II) were formed by casting CTA from the organic solution containing phosphonium ionic liquid as ion carrier and nitrophenyloctyl ether (ONPOE) to form a thin, stable film. The selectivity and efficiency of the facilitated transport of metal ions from an aqueous solution through PIMs depend on the extraction capability of the carrier. The kind of carrier in the membrane plays a significant role during the permeation of metal ions in transport processes. High process selectivity is achieved when an appropriate carrier is used for synthesis of the membrane. Therefore, the choice and the application of an appropriate ion carrier is very important. To study the influence of the type of carrier in PIM on the facilitated transport process of Cd(II) and Cu(II) ions, the PIMs were prepared by using two quaternary phosphonium salts: Cyphos IL 101 and Cyphos IL 104. The ionic liquids contain different anions. The resulting membrane contained 15.6 wt.% CTA1 and 41.2 wt.% ion carrier and 43.2 wt.% ONPOE. Cyphos IL 101 and Cyphos IL 104 have similar densities (*d*_Cyphos 101_ = 0.880 g·cm^−3^ and *d*_Cyphos 104_ = 0.892 g·cm^−3^). One of the main aims of this work is to study the transport kinetics of Cd(II) and Cu(II) across PIMs with Cyphos IL 101 and Cyphos IL 104. The compounds have been chosen as the ion carriers in CTA membranes on the basis of the preliminary studies on solvent extraction.

**Fig. 3:**
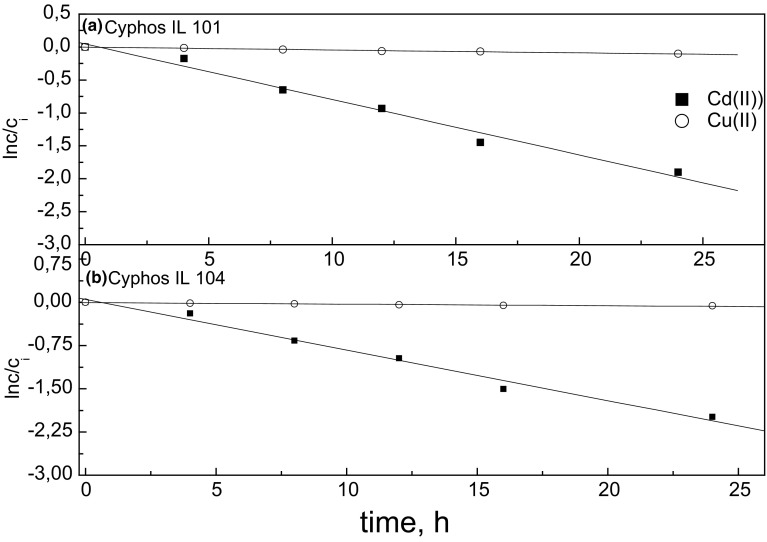
Plot of ln(*c*/*c*_*i*_) versus time for Cd(II) and Cu(II) transport across PIM depending on the kind of ion carrier. PIM: 0.075 g CTA, 2.7 cm^3^ ONPOE/1 g CTA, 1.5 mol·dm^−3^ ion carrier; the source phase: 0.01 mol·dm^−3^ Cd(II), Cu(II) in 0.1 mol·dm^−3^ HCl; the receiving phase: 1 mol·dm^−3^ H_2_SO_4_

In preliminary experiments, the transport studies of Cd(II) and Cu(II) were carried out from 0.1 mol·dm^−3^ HCl as the source solution into 1 mol·dm^−3^ H_2_SO_4_ through PIMs containing Cyphos IL 101 or Cyphos IL 104 as the selective ion carriers. We can expect that anions of the ILs will affect on the permeability of the studied cations. Figure 3 shows the kinetic dependence of ln(*c*/*c*_*i*_) versus time for the transport of Cd(II) and Cu(II) through PIMs with different ion carriers. Table 2 shows the variation of the rate constants, the initial fluxes, recovery factors (*RF*) of Cd(II) and Cu(II) after 24 h and the selectivity coefficients (*S*_Cd/Cu_) versus the type of ion carrier in the membrane. The results show that the initial fluxes of metal ions through PIMs with different carriers decrease in the sequence: Cd(II) > Cu(II). This means that the anion in the structure of the studied phosphonium ILs does not change the order of selectivity of Cd(II) over Cu(II) from 0.1 mol·dm^−3^ HCl. However, the higher selectivity coefficient (*S*_Cd/Cu_) was obtained for PIM with Cyphos IL 104 as the ion carrier. We observe that the recovery factors and initial fluxes of Cu(II) through PIMs with both phosphonium ILs are very low, making the transport selectivity of Cd(II) over Cu(II) ions very high. The initial fluxes of Cd(II) and Cu(II) are 18.8 and 0.67 μmol·m^−2^·s^−1^, respectively, for the membrane containing Cyphos IL 104 as ion carrier. As can be seen from Table 2, the recovery factor of Cd(II) was also the highest (above 86 %) for PIM with Cyphos IL 104. Thus, the PIM with Cyphos IL 104 as carrier was used in the next series of experiments.

**Table 2:** Kinetic parameters for Cd(II) and Cu(II) transport across the PIM depending on the kind of ion carrier

Ion carrier	Rate constant *k* (h^−1^)		Initial flux *J*_*i*_ (μmol·m^−2^·s^−1^)		Recovery factor (%) (after 24 h)		Selectivity coefficient *S*_Cd/Cu_
	Cd(II)	Cu(II)	Cd(II)	Cu(II)	Cd(II)	Cu(II)	
Cyphos IL 101	0.084	0.004	18.0	0.97	85.1	9.7	18.5
Cyphos IL 104	0.085	0.003	18.8	0.67	86.3	5.9	28.1

PIM: 0.075 g CTA, 2.7 cm^3^ ONPOE/1 g CTA, 1.5 mol·dm^−3^ ion carrier; the source phase: 0.01 mol·dm^−3^ Cd(II), Cu(II) in 0.1 mol·dm^−3^ HCl; the receiving phase: 1 mol·dm^−3^ H_2_SO_4_

### Effect of Hydrochloric Acid Concentration in the Source Phase

**Fig. 4:**
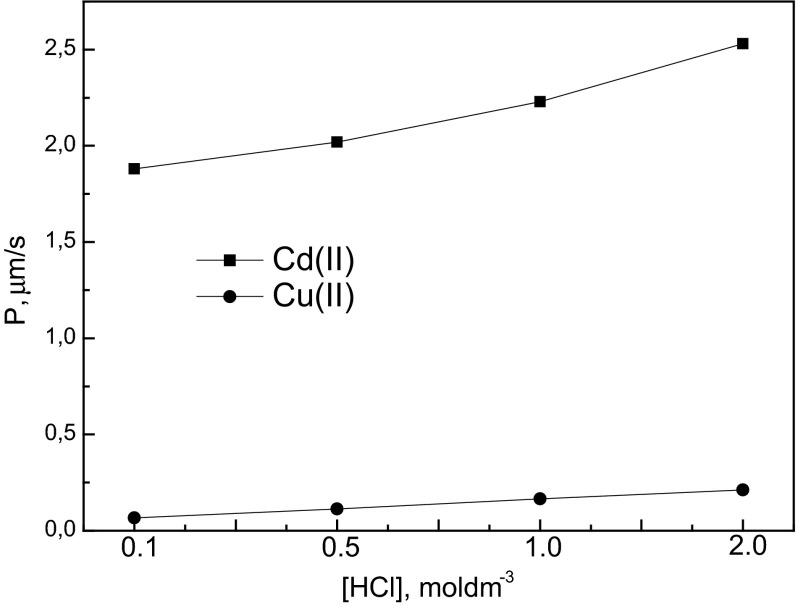
Effect of HCl concentration in the source phase on the permeability coefficient (*P*) of Cd(II) and Cu(II) across PIM with Cyphos IL 104. PIM: 0.075 g CTA, 2.7 cm^3^ ONPOE/1 g CTA, 1.5 mol·dm^−3^ Cyphos IL 104; the source phase: 0.01 mol·dm^−3^ Cd(II), 0.01 mol·dm^−3^ Cu(II) in HCl; the receiving phase: mol·dm^−3^ H_2_SO_4_

Cyphos IL 104 was chosen as the selective ion carrier on the basis of the solvent extraction results and the preliminary PIM experiments. The ion carrier was physically immobilized in the polymer matrix containing ONPOE, which was used as the plasticizer as well as solvent for carrier. Hydrochloric acid solutions were used to determine the effect of the acid concentration in the source phase on Cd(II) and Cu(II) transport within the PIMs system. In order to investigate the influence of hydrochloric acid concentration in the source phase on the transport of metal ions, the experiments were performed at HCl concentrations varying from 0.1 to 2.0 mol·dm^−3^. Separation of Cd(II) and Cu(I) was carried out from the source phase containing 0.01 mol·dm^−3^ of each metal ion; 1 mol·dm^−3^ H_2_SO_4_ was used as the receiving phase. Figure 4 shows the dependence of the permeability coefficient (*P*) of the cations transported on the HCl concentration. As can be seen from this figure, the permeability coefficient of Cd(II) increased with HCl concentration in the source phase from 1.88 μm·s^−1^ at 0.1 mol·dm^−3^ HCl to 2.53 μm·s^−1^ at 2 mol·dm^−3^ HCl. The dependence for the transport of Cu(II) is very similar but the increase of the permeability coefficient values is slightly lower. As can be seen from this figure, the permeability coefficient of Cu(II) increased with HCl concentration, increasing from 0.067 μm·s^−1^ at 0.1 mol·dm^−3^ HCl to 0.211 μm·s^−1^ at 2 mol·dm^−3^ HCl as the source phase. It appears that an increase in hydrochloric acid concentration is responsible for an increase in the permeability coefficient of Cd(II), likely due to the presence of anionic species, mainly ${\text{CdCl}}_{3}^{ - }$ in the source phase. The permeability coefficients calculated for Cd(II) and Cu(II) transport from the source phase of various concentrations of hydrochloric acid confirm that PIM transport depends strongly on the acid and chloride ion concentrations in the source phase.

A mathematical model can be proposed in this study for the explanation of permeation kinetics of Cd(II) and Cu(II). This model takes into account both the diffusion of Cyphos IL 104 and its ion pair with the chlorocomplexes of the metal ions within the PIM and the kinetics of the interfacial reaction. We can determine the content of metal species in the aqueous chloride solutions on the basis of the theoretical values of chlorocomplexes formation. Table 3 shows species of Cd(II) and Cu(II) in the aqueous chloride solutions, calculated from data on chloro-complex stability constants from the literature. The literature values of stability constants for Cd(II) are the following: log_10_*β*_1,Cd_ = 1.46; log_10_*β*_2,Cd_ = 1.95; log_10_*β*_3,Cd_ = 2.17 [18, 35]. The values for the Cu(II) complexes are: log_10_*β*_1,Cu_ = 0.93; log_10_*β*_2,Cu_ = 0.79; log_10_*β*_3,Cu_ = 0.46; log_10_*β*_4,Cu_ = 0.01 [35]. Table 3 shows the percent molar contributions of chloride complex species (%) for Cd(II) and Cu(II). As can be seen from this table, in the case of Cd(II), the predominant species of Cd(II) at 0.1 mol·dm^−3^ Cl^−^ is CdCl^+^, at 0.5 mol·dm^−3^ Cl^−^ it is CdCl_2_ and at 1–2 mol·dm^−3^ Cl^−^ it is ${\text{CdCl}}_{3}^{ - }$. Therefore, it can be concluded that the permeation of Cd(II) is better in the presence of a high concentration of chloride ions and anionic chlorocomplexes of Cd(II), which are more easily transported from the source phase. A similar dependence in the transport of Cd(II) through PIM with Cyphos IL 104 was obtained in the previous study [18]. It can be observed that permeation was very effective for the transport of Cd(II) from sodium chloride solution as well as from hydrochloric acid solutions, with similar values. On the other hand, the predominant species of Cu(II) at 0.1 mol·dm^−3^ Cl^−^ is Cu^2+^, at 0.5–1 mol·dm^−3^ Cl^−^ is CuCl^+^ and at 2 mol·dm^−3^ Cl^−^ CuCl_2_ (Table 3). The results obtained from the transport process show that permeation of Cu(II) was not improved by increasing chloride concentration. The transport of Cd(II) across the PIM with Cyphos IL 104 is much faster than for Cu(II), which can be explained by the much higher extraction constant of Cd(II). Wang et al. [19] reported a similar observation in the transport of Cd(II) and Cu(II) from HCl solutions through PIM with Aliquat 336 (trioctylmethylammonium chloride), which is also an ionic liquid, quaternary ammonium salt.

**Table 3:** The percent molar contributions of chloride complex species (%) for Cd(II) and Cu(II) depending on chloride ion concentrations

Metal	[Cl^−^] (mol·dm^−3^)	M^2+^ (%)	MCl^+^ (%)	MCl_2_ (%)	${\text{MCl}}_{3}^{ - }$ (%)	${\text{MCl}}_{4}^{2 - }$ (%)
Cd(II)	0.1	12.3	51.4	31.2	5.1	–
	0.5	1.9	26.3	52.1	19.7	–
	1.0	–	8.5	44.3	47.2	–
	2.0	–	2.8	36.1	61.1	–
Cu(II)	0.1	52.6	43.7	3.7	–	–
	0.5	16.1	58.4	20.3	5.2	–
	1.0	5.6	42.8	31.6	16.3	3.7
	2.0	3.9	11.5	32.4	29.1	23.1

**Fig. 5:**
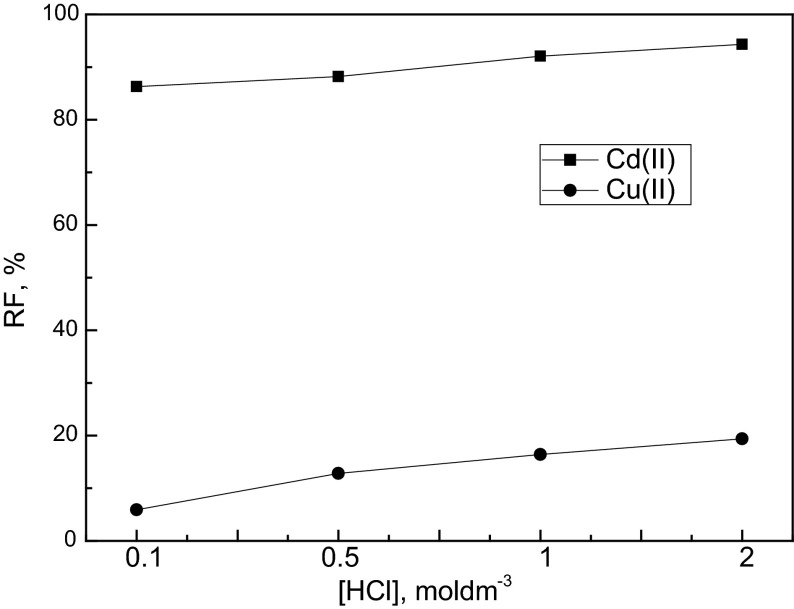
Relation of recovery factor [*RF* (%)] of Cd(II) and Cu(II) after 24 h versus HCl concentration in the source phase. Experimental conditions as in Table 3

Table 4 shows the kinetic parameters of this transport processes. It was observed that the initial fluxes of Cd(II) and Cu(II) achieved the highest values of 25.3 and 2.11 μmol·m^−2^·s^−1^, respectively, from 2 mol·dm^−3^ HCl as the source phase. The dependence on the recovery factor (%) of Cd(II) and Cu(II) ions from HCl solutions as the function of the acid concentration is shown in Fig. 5. It was observed that the recovery factor of Cd(II) and Cu(II) transport achieved the highest values of 94.3 and 19.4 %, respectively, at 2 mol·dm^−3^ HCl in the source phase. As can be observed from Table 3, the selectivity coefficient for Cd(II) over Cu(II) decreases with HCl concentration increasing in the source phase from 28.1 for 0.1 mol·dm^−3^ HCl to 11.2 for 2 mol·dm^−3^ HCl. Guo et al. [31] reported also the effect of HCl concentration in the source phase on Cr(VI) transport through PIM with Cyphos IL 104 and obtained a similar dependence. They observed that with HCl concentration increasing in the feed phase, the initial flux of Cr(VI) also increased. Comesana et al. [34] showed the transport of Cd(II) from HCl solution by non-dispersive solvent extraction with strip dispersion (NDSXSD) using Cyphos IL 101. They observed decreasing Cd(II) permeation when the hydrochloric acid concentration of the source phase is decreased.

**Table 4:** The initial fluxes, rate constants and selectivity coefficients for competitive transport of Cd(II) and Cu(II) across PIM with Cyphos IL 104

[HCl] (mol·dm^−3^)	Rate constant *k* (h^−1^)		Initial flux *J*_*i*_ (μmol·m^−2^·s^−1^)		Selectivity coefficient *S*_Cd/Cu_
	Cd(II)	Cu(II)	Cd(II)	Cu(II)	
0.1	0.085	0.003	18.8	0.67	28.1
0.5	0.091	0.005	20.2	1.13	17.9
1	0.101	0.007	22.3	1.65	13.5
2	0.114	0.009	25.3	2.11	11.2

PIM: 0.075 g CTA, 2.7 cm^3^ ONPOE/1 g CTA, 1.5 mol·dm^−3^ Cyphos 104; the source phase: 0.01 mol·dm^−3^ Cd(II), 0.01 mol·dm^−3^ Cu(II) in HCl; the receiving phase: 1 mol·dm^−3^ H_2_SO_4_

### Effect of Chloride Ion Concentration in the Source Phase

**Fig. 6:**
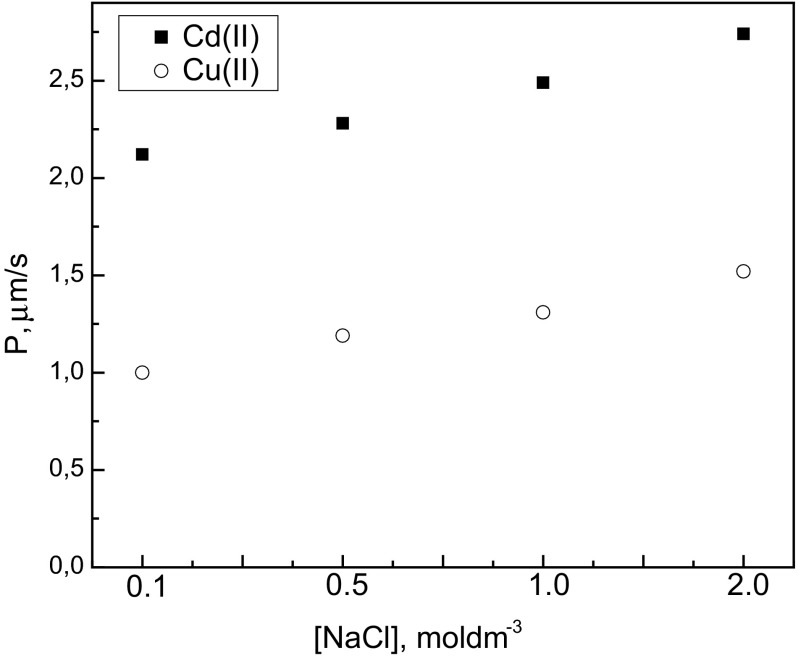
The permeability coefficient (*P*) of Cd(II) and Cu(II) versus chloride concentration in the source phase from NaCl solutions. PIM: 1.5 mol·dm^−3^ Cyphos IL 104, 2.7 cm^3^ ONPOE/1 g CTA; the source phase: 0.01 mol·dm^−3^ Cd(II) and 0.01 mol·dm^−3^ Cu(II); the receiving phase: 1 mol·dm^−3^ H_2_SO_4_

The influence of chloride ion concentration on the initial flux (*J*_*i*_) of Cd(II) and Cu(II) was investigated. The experiments were carried out at various chloride concentrations provided by sodium chloride. The concentration of chloride ions [Cl^−^] was varied between 0.1 and 2 mol·dm^−3^. The results are presented in Fig. 6. This figure shows changes of the permeability coefficients of metal ions with chloride concentration in the source phase. As can be observed from this figure, when the chloride ions concentration increases, the values of the permeability coefficients also increase for both metal ions. The permeability coefficients of Cd(II) and Cu(II) reached the highest values of 2.75 and 1.52 μm·s^−1^, respectively, at 2 mol·dm^−3^ chloride in the source solution. It can be observed that the transport of Cd(II) was very effective from highly saline media. Similar dependences in the transport of Cd(II) and Cu(II) through the PIM with Cyphos IL 104 were obtained from hydrochloric acid solutions but the obtained values of permeability coefficients are higher in the case of transport of metal ions from sodium chloride solutions. We can see that permeation was more effective for the transport of Cd(II) than Cu(II) from sodium chloride solutions as well as from hydrochloric acid solutions.

**Fig. 7:**
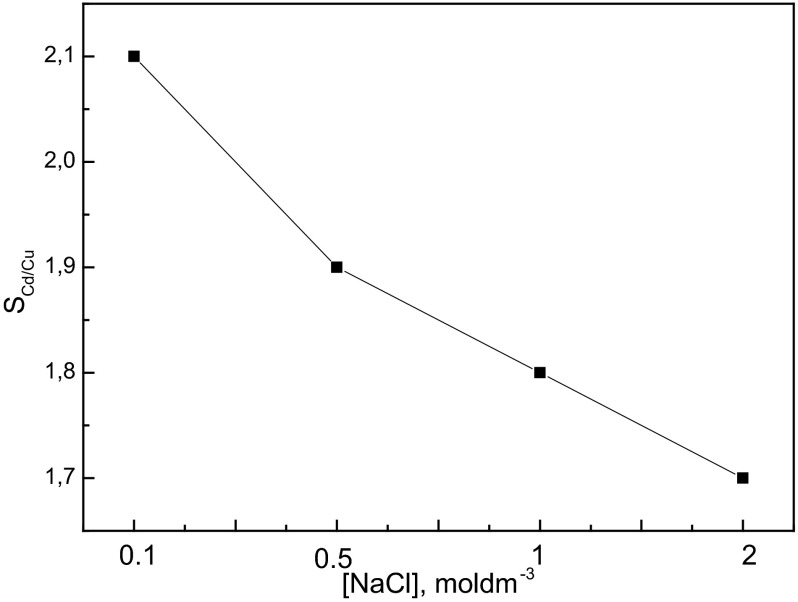
The selectivity coefficient of Cd(II) over Cu(II) versus NaCl concentration. Experimental conditions as in Fig. 6

Figure 7 presents the selectivity of this transport process. As can be seen, the selectivity coefficient (*S*_Cd/Cu_) of Cd(II) over Cu(II) decreases in neutral medium when the chloride ions concentration increases. The highest separation coefficient (*S*_Cd/Cu_) was obtained at the lowest sodium chloride concentration, i.e., 0.1 mol·dm^−3^ NaCl. The successful separation of metal ions across the membrane is dependent on the chloride ions concentration in the source phase and chemical forms of metal ion in this solution. Adelung et al. [36] and Upitis et al. [20] also studied the separation of Cd(II) and Cu(II) from chloride solutions by transport across PIM with the quaternary ammonium salt—Aliquat 336. They reported that the formation of negatively charged metal chloride complex species was responsible for the extraction of the target metal to PIMs. Experimental results further confirm that the transport selectivity between Cd(II) and Cu(II) can be controlled by regulating the chloride concentration of the source phase.

## Conclusions

Results of the presented investigations prove that Cyphos IL 101 and Cyphos IL 104 in toluene can be successfully used as the extractants for Cd(II) removal from chloride media. Extraction of Cd(II) is very fast and efficient (over 99 %). The presence of HCl in the feed enhances Cd(II) and Cu(II) extraction and decreases the selectivity coefficient for Cd(II) over Cu(II). The obtained results allow the conclusion that the transport process across PIMs with Cyphos IL 104 as the ion carrier provides an attractive alternative to conventional solvent extraction methods for the separation of Cd(II) and Cu(II) from hydrochloric acid solutions. The studied PIMs with phosphonium ILs can be recommended for the separation of Cd(II) and Cu(II) from hydrochloric acid solutions with regard to the high values of separation coefficients. The higher selectivity coefficient of Cd(II) over Cu(II) from 0.1 mol·dm^−3^ HCl as the source phase, was obtained using membranes with Cyphos IL 104. It was found that the initial fluxes of Cd(II) and Cu(II) are the highest for 2 mol·dm^−3^ HCl as the source solution and are 25.3 and 2.11 μmol·m^−2^·s^−1^, respectively. However, the separation coefficients decrease with increasing HCl concentration in the source phase, from 28.1 at 0.1 mol·dm^−3^ HCl to 11.2 at 2 mol·dm^−3^ HCl. The selectivity of this metal ions transport decreased also in neutral media when the chloride ions concentration increased. The transport process through the PIM containing 15.6 wt.% CTA and 41.2 wt.% Cyphos IL 104 and 43.2 wt.% ONPOE enabled extraction above 86 % of Cd(II) from 0.1 mol·dm^−3^ hydrochloric acid into 1 mol·dm^−3^ H_2_SO_4_ after 24 h. Finally, the separation membrane system presented in this paper can be useful for the separation of Cd(II) and Cu(II) from hydrochloric acid solutions.
